# Collective Emotions and Social Resilience in the Digital Traces After a Terrorist Attack

**DOI:** 10.1177/0956797619831964

**Published:** 2019-03-13

**Authors:** David Garcia, Bernard Rimé

**Affiliations:** 1 Section for Science of Complex Systems, Center for Medical Statistics, Informatics and Intelligent Systems, Medical University of Vienna; 2 Complexity Science Hub Vienna, Vienna, Austria; 3 Psychological Sciences Research Institute, University of Louvain

**Keywords:** social interaction, psycholinguistics, emotions, social media, open data

## Abstract

After collective traumas such as natural disasters and terrorist attacks, members of concerned communities experience intense emotions and talk profusely about them. Although these exchanges resemble simple emotional venting, Durkheim’s theory of collective effervescence postulates that these collective emotions lead to higher levels of solidarity in the affected community. We present the first large-scale test of this theory through the analysis of digital traces of 62,114 Twitter users after the Paris terrorist attacks of November 2015. We found a collective negative emotional response followed by a marked long-term increase in the use of lexical indicators related to solidarity. Expressions of social processes, prosocial behavior, and positive affect were higher in the months after the attacks for the individuals who participated to a higher degree in the collective emotion. Our findings support the conclusion that collective emotions after a disaster are associated with higher solidarity, revealing the social resilience of a community.

Individual responses to collective traumas (e.g., natural disasters, technological accidents, terrorist attacks) have been abundantly investigated. In particular, national surveys conducted in the United States after the September 2001 attacks (e.g., Seery, Silver, Holman, Ence, & Chu, 2008; Silver, Holman, McIntosh, Poulin, & Gil-Rivas, 2002; Stein et al., 2004) have extensively documented the implications of exposure to such episodes for individuals’ psychological adjustment. Collective traumas also spark spectacular collective manifestations (Collins, 2004b; Pennebaker & Harber, 1993) about which much less is known. A particularly visible collective response to upheavals takes the form of crowd gatherings, such as marches, demonstrations, copresence at memorial sites, or formal commemorations. After September 2001, countless gatherings happened all over the United States (Collins, 2004b). After the Madrid attacks in March 2004, protest marches took place in all Spanish cities, with an estimated 25% of the population taking part (Páez, Basabe, Ubillos, & González-Castro, 2007). The attacks on Charlie Hebdo in Paris in January 2015 provoked spontaneous marches that brought together 4 million to 5 million demonstrators across France (“*Contre le Terrorisme*,” 2015).

A much less noticeable though equally important social response to upheavals happens in interpersonal communication. Because he witnessed the 1910 San Francisco earthquake, William James was probably the first to document the extraordinary need to talk among disaster survivors. In his correspondence with Pierre Janet, he reported that in rescue tents, it was impossible to sleep at night because of the endless chatter (Janet, 1926/1975, p. 326). After a traumatic event, members of concerned communities indeed talk profusely about what happened and how they feel. Pennebaker and Harber (1993) provided the first quantitative empirical evidence in this respect. In surveys conducted respectively among residents of the San Francisco Bay Area after the 1989 earthquake and among U.S. respondents after the outbreak of the first Gulf War, they recorded startling levels of social sharing that lasted for a period of 2 to 3 weeks after the event but dropped sharply afterward. Similarly, after the 2004 terrorist attacks in Madrid, Rimé, Páez, Basabe, and Martínez (2010) observed that Spanish residents talked and heard talking of the event at a very high level during the 1st week following the attacks. The level of talking decreased during the 2nd and 3rd weeks, and 2 months after the event, the social sharing of emotion had vanished.

Such communications were virtually untraceable before the development of our current digital society; social media now reveal the overabundant communications provoked by upheavals (for a review, see Simon, Goldberg, & Adini, 2015). After the 2011 earthquake in Japan, the volume of tweets sent per second spiked to more than 5,000 (abdur, 2011). After the January 2015 terrorist attacks in Paris, the hashtag #JeSuisCharlie was used nearly 6,500 times per minute (Goldman & Pagliery, 2015). To date, we lack scientific knowledge on the nature and function of these profuse communications in the midst of collective traumas. At first sight, they look like buzzing noise stimulated by transient ventilation needs and devoid of noteworthy social consequences. We advocate in favor of a radically different perspective. We propose that crowd gatherings and peer-to-peer communications elicited by upheavals rest on common ground. In our perspective, they involve similar processes of social synchronization and they entail effects serving social resilience. We first develop our arguments and then describe a study designed to assess our view.

## Collective Gatherings, Synchronization, and Social Resilience

Durkheim (1912) theorized that members of a society happen to come together and to synchronize their thoughts and actions through shared slogans, gestures, and movements. Participants reciprocally stimulate their emotions, thus engendering a collective effervescence. In this experience of emotional communion, they revive their sense of social belonging and their shared beliefs. Individuals thus leave the crowd endowed with positive affect, strength and self-confidence, and openness to other individuals. Recent studies largely supported this classic model. First, experimental induction of synchronization (e.g., marching in step, dancing in rhythm) lessened interpersonal boundaries, enhanced positive affect, and promoted cooperation (e.g., Miles, Nind, Henderson, & Macrae, 2010; Wiltermuth & Heath, 2009). Second, participation in both positively and negatively toned collective gatherings strengthened common identity, personal and collective self-esteem, positive affect, and positive social beliefs (Páez, Rimé, Basabe, Wlodarczyk, & Zumeta, 2015). In line with a central tenet of Durkheim’s model, these effects were mediated by participants’ perceived emotional synchrony with other people. The question thus arises of the social functions of social synchronization and its effects. Cacioppo, Reis, and Zautra (2011) stressed that coordinated social activity, feelings of connectedness, and shared identities represent key contributions to social resilience or the capacity to “endure and recover from life stressors” (p. 44). Similarly, disaster-preparedness models focus on social connectivity, or bonding, bridging, and linking, as a primary asset within resilient communities (Ellis & Abdi, 2017). In sum, social synchronization may favor social resilience.

## Social Sharing of Emotion, Synchronization, and Social Resilience

The profuse talking of members of affected communities simply fits the principle of social sharing of emotion. It states that emotion systematically triggers the need to talk about it and that more intense emotions are shared more frequently and more extensively (for a review, see Rimé, 2009). However, collective emotional episodes are special in that they elicit emotion sharing feedback loops. Individuals read news, think of the event, talk, and hear other people talking about it (Collins, 2004b; Pennebaker & Harber, 1993; Rimé, 2007). As sharing reboots emotions (Rimé, 2009), both the sharing source and the target experience further need to talk. In the affected community, with most individuals being the seat of this process, the social sharing of emotion can reach astronomic values.

To our knowledge, no theoretical model to date accounts for this spectacular peer-to-peer sharing of collective events. Moving from a peer-to-peer perspective to a collective perspective on these exchanges, we propose that even if participating individuals remain physically separated from one another, the social sharing of a collective emotion drives social synchronization in the affected community and thus fuels a variant of Durkheim’s collective effervescence. We argue that the buzzing noise elicited by a collective emotional event actually weaves a social resilience process based on social synchronization. More specifically, we predict participation in the social sharing of emotion following a traumatic event to enhance social belonging, shared beliefs, positive affect, and prosocial attitudes. Initial support for such predictions was found in longitudinal data collected from respondents’ self-reports after the March 2004 terrorist attacks in Madrid (Rimé et al., 2010). One week, 3 weeks, and 8 weeks after the terrorist act, respondents from five Spanish regions answered questionnaires measuring social sharing of emotion related to the events as well as perceived emotional climate, social integration, and posttraumatic growth. Higher levels of social sharing were initially found to predict higher levels of social integration, perceived posttraumatic growth, and positive affect assessed 3 and 8 weeks later. This was further linked to perceived benefits after the terrorist attacks (Vázquez & Hervás, 2010).

Nowadays, digital traces from social media in combination with text analysis offer the opportunity to access emotional life at a much larger scale than using self-reports (Schweitzer & Garcia, 2010). Cohn, Mehl, and Pennebaker (2004) analyzed the content of online diaries of U.S. users 2 months before and 2 months after September 11, 2001. Participants expressed more negative emotions and were more cognitively and socially engaged within the first 2 weeks after the attacks but then returned to baseline levels. Currently, Twitter offers direct access to individuals’ emotional sharing content. Eriksson (2016) analyzed discourse on Twitter in the 6 days following the 2011 attacks in Oslo and Utøya. Initially, the main discursive themes included solidarity, but later, the causes of the attacks were framed with respect to national contexts. Smith, McGarty, and Thomas (2018) investigated tweets posted in reaction to the diffusion of images of the body of 3-year-old Aylan Kurdi. Tweeting about death, threat, and harm predicted increased expressions of solidarity with refugees 10 weeks later.

Beyond these effects, linguistic analysis of observational data revealed how language patterns change in reaction to economic recessions and wars (Iliev, Hoover, Dehghani, & Axelrod, 2016) and how they vary across levels of activity in a political movement (Alvarez, Garcia, Moreno, & Schweitzer, 2015). Thus, studies examining digital traces surrounding major upheavals confirmed the abundant emotional, cognitive, and social engagement in online communicative exchanges. Twitter has become a go-to site during disasters and a way to share methods for donating money to help the recovery (for a review, see Simon et al., 2015). The terrorist attacks in Paris in November 2015 led to expressions of solidarity and empathy across the globe through the #JeSuisParis hashtag. At the same time, inhabitants of Paris were actively using the hashtag #PorteOuverte to offer their homes to people who were afraid or unable to travel inside Paris in the aftermath of the attacks. This was a clear example of prosocial behavior as manifested through social media: Users were not just talking about helping other people; they were actually offering help through Twitter in an altruistic act toward strangers.

In the present study, we empirically analyzed collective effervescence, testing the association between the collective experience of emotions and solidarity, in particular the activation of social processes and the salience of prosocial behavior and shared values (Collins, 2004a). We present the analysis of French social media content on Twitter after the November 2015 terrorist attacks in Paris. We analyzed the temporal dynamics of the collective emotion that followed the attacks and tested its relationship to lexical indicators associated with solidarity.

## Method

### Twitter data set

During 1 month after November 13, 2015, we collected tweets with hashtags related to the terrorist attacks in Paris through daily requests to the Twitter search application programming interface (API). Hashtags were selected by manual inspection to cover tweets referring to the attacks, and a list of them can be found in the Supplemental Material available online. In the results of those requests, we filtered out all tweets that were retweets, and we applied language detection to each tweet in combination with language metadata provided by Twitter to select only tweets in French. Analyzing tweets sampled through manually selected hashtag lists can lead to unrepresentative samples of Twitter data (see King, Lam, & Roberts, 2017, who include an example about the Paris attacks in their Appendix). Using only that data would have limited the analysis only to the days after the attacks, ignoring earlier periods and artificially lowering data volumes over time. Instead of using those hashtag tweets as a corpus, we used them to identify a panel of user accounts that we historically analyzed through their time line of original tweets (ignoring retweets).

**Fig. 1.**
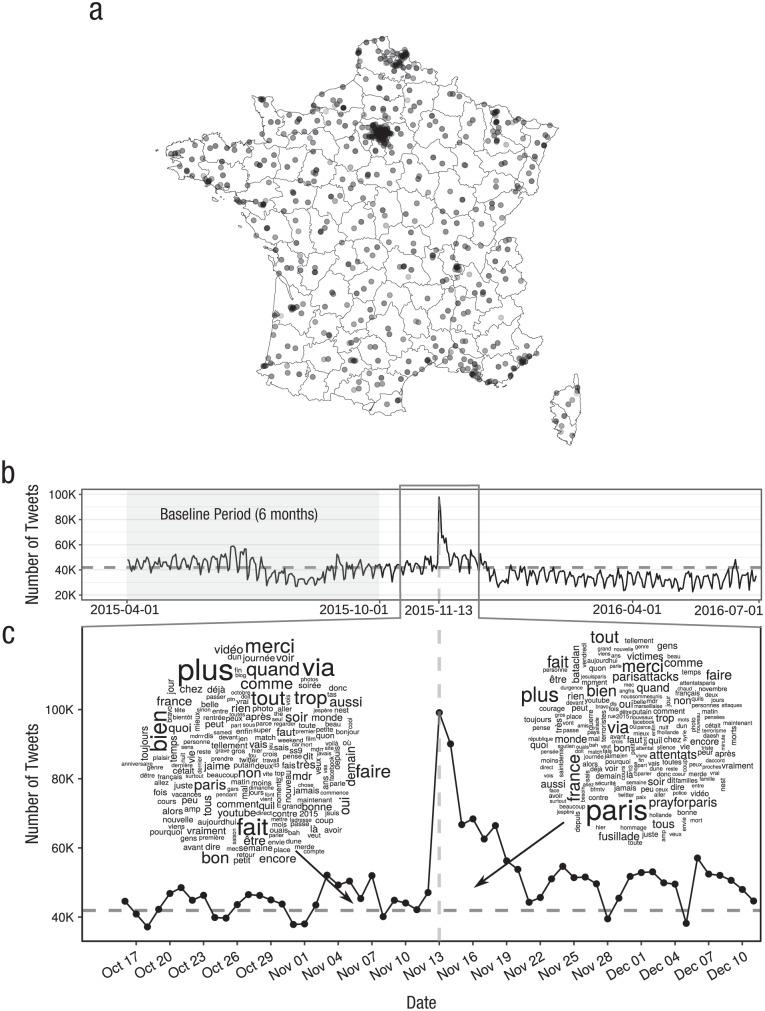
Twitter data around the Paris terrorist attacks of November 2015. Locations of the Twitter users in our data set are shown on a map of France (a), with points shaded according to the amount of data available in each location (darker equals more data). The full time line (b) shows the number of tweets in the data set, and the focus window of our analysis is expanded below (c). Word clouds show words with a size proportional to their frequency in the days before and after the attacks. In (b) and (c), dashed horizontal lines mark the average daily number of tweets, and the vertical line marks the day of the attacks. The shaded area in (b) shows the data used to calculate linguistic baselines.

Of the nearly half million users found in that data set, more than 287,000 (58%) shared location information in their profile. We filtered those users to include only those with profiles located in European France and who showed no clear signs of behaving like bots or mass media accounts (for more details, see the Supplemental Material). As a result, we produced a data set of 62,114 user accounts in the country directly affected by the terrorist attacks.^1^ The activity of users in this data set was concentrated in Paris but also covered locations all over France, as shown in Figure 1a. We restricted our final data set to the period between April 1, 2015, and June 30, 2016, shown in Figure 1b, which comprised a total of 17,899,591 tweets. There was a clear activity spike following the attacks, when the number of tweets was more than double any previous day in the data set. A qualitative inspection of the text of these tweets, as shown in Figure 1c, shows that tweets after the attacks often included terms such as *Paris, prayforparis*, and *attentats* (French for “terrorist attacks”). This illustrates that the activity spike was directly related to the attacks, motivating our further quantitative analysis of the text of tweets.

### Text analysis

We processed all tweets using the Linguistic Inquiry and Word Count (LIWC) method (Pennebaker, Boyd, Jordan, & Blackburn, 2015), computing lexical indicators as the fraction of words in a text that belong to a word class of LIWC. Using the French adaptation of the LIWC dictionary (Piolat, Booth, Chung, Davids, & Pennebaker, 2011), we measured the frequency of use of positive-affect and negative-affect terms in tweets as well as the expression of terms related to sadness, anxiety, and anger. We validated the applicability of the French LIWC dictionary with a data set of French tweets with annotated sentiment. Compared with control tweet samples, tweets annotated as positive contained 110% more positive-affect terms, and tweets annotated as negative contained 420% more negative-affect terms. Furthermore, tweets annotated as sad contained 933% more sadness terms, tweets annotated as anxious contained 1,150% more anxiety terms, and tweets annotated as angry contained 360% more anger terms. These results are reported in detail in the Supplemental Material.

We additionally recorded the fraction of terms in the LIWC category of social processes, which contains terms that refer to other people (*somebody, guy*), relationships ( *friend, son*), and social activities (*discuss, celebration*). Furthermore, we quantified the frequency of terms about French shared values (stemming from the terms *liberté, égalité*, and *fraternité*). Finally, we measured the frequency of terms related to prosocial behavior through a translation of a previously validated dictionary (Frimer, Schaefer, & Oakes, 2014), including terms such as *caring, solidarity*, and *NGO* (nongovernmental organization). We validated our French version of the prosocial-terms dictionary by comparing a sample of prosocial Twitter accounts with a random sample of tweets from our baseline data set. Tweets from prosocial Twitter accounts contained 156% more prosocial terms than tweets in the baseline data set (see the Supplemental Material).

### Statistical models

We calculated the daily mean value of each of the above lexical indicators over the tweets produced during our analysis period. We computed the baseline of each indicator for each weekday during the 6-month period before the attacks, shown in Figure 1b. Then, for each indicator, we computed the daily normalized score as the log-transformed fraction between the value of the indicator in a day and the corresponding baseline value according to the day of the week. In this way, we corrected for weekly oscillations in term frequencies (Golder & Macy, 2011), reduced skewness, and produced comparable scores for all indicators. To assess the uncertainty of this measurement, we generated 10,000 bootstrapped samples of the tweets in each day to calculate 95% confidence intervals (CIs) and median estimates.

We fitted the temporal evolution of each daily score through a time-series model that included an exogenous shock when the attacks took place and a memory term (ϕ) that quantified the linear relationship between the score in one day and the score in the next day. The value of ϕ is a measurement of the memory of the time series and tells us about the strength of synchronization of behavior in the aftermath of the terrorist attacks. Significant positive values of ϕ are generated when there is strong alignment of behavior that creates collective dynamics beyond individual relaxation levels. A value of ϕ indistinguishable from zero indicates the absence of collective behavior, which is present when synchronization is absent or weaker than individual relaxation. We illustrate this in detail in the Supplemental Material through an agent-based model of collective emotions (Schweitzer & Garcia, 2010) that was calibrated with empirical results on emotion dynamics in online discussions (Garcia, Kappas, Küster, & Schweitzer, 2016).

We fitted models of collective behavior with the bayesglm function of the *arm* package (Gelman & Su, 2018) in the R programming environment, taking weakly informative priors for all parameters. After fitting, we verified that all residuals approximately followed normal distributions with Shapiro-Wilk tests, assessed that residuals of time-series models had no significant serial correlations in stationarity tests, and verified that they showed no relevant signs of heteroscedasticity. Detailed results of all fits are reported in the Supplemental Material.

Our individual-level analysis tested whether there was a relationship between high levels of emotionality right after the attacks and long-term increased levels of social-process terms, prosocial terms, shared-values terms, and affect terms. This analysis was restricted to users from our sample who produced at least one tweet in the 3 months before the attacks (August 13–November 12), in the 2 weeks after the attacks, and in the next 3 months, resulting in a total of 49,001 users. Because we sought to study the effect of shared emotions soon after the attacks on long-term behavior, we measured the individual level of emotional expression in the 2 weeks after the attacks (November 13–27) and analyzed its effect on other lexical indicators measured in the next 3 months (November 28–February 27).

For each user, we first calculated a vector of personality-related lexical indicators in the 3 months before the attacks. The digital traces of human behavior capture a wide variety of behaviors commonly studied in personality research, such as religious faith, caring, and empathy (Boyd et al., 2015). Our vector of personality-related lexical indicators included positive-affect and negative-affect terms, social-process terms, and first-person singular pronouns, which have been found to be correlated with extraversion and openness (Mairesse, Walker, Mehl, & Moore, 2007), especially in social media (Schwartz et al., 2013). This allowed us to correct for personality components that are likely to play a role in the susceptibility of a person to participate in a collective emotion. To take into account self-selection biases, we regressed emotional expression after the attacks on the vector of personality-related lexical indicators, adding to the model the logarithm of the number of posts of each user to account for different user-engagement levels. This model indicates significant correlations between emotional expression after the attacks and all indicators except social processes (for more detail, see the Supplemental Material). For the rest of our analysis, we took the residuals of this model as the corrected measurement of short-term emotionality after the attacks.

We measured long-term effects in social-process terms, prosocial terms, shared values, and affect during the 3-month period after the attacks. We estimated effect sizes in mediation analysis using the *mediation* package in R (Tingley, Yamamoto, Hirose, Keele, & Imai, 2014), taking as the mediator the measurement of the same lexical indicator but in the 2 weeks after the attacks. This allowed us to measure direct effects in long-term measurements versus mediated effects through the short-term levels of the dependent variable. We also added personality-related lexical indicators measured during the baseline period as covariates in each model. We assessed the uncertainty of these estimates in 10,000 bootstrapped samples, using the 25th and 75th percentiles of the corrected measurement of emotionality after the attacks to estimate the effect on the long-term levels of use of solidarity and affective terms. Detailed results of all fits and effect-size estimates are reported in the Supplemental Material.

## Results

### Collective behavior

**Fig. 2.**
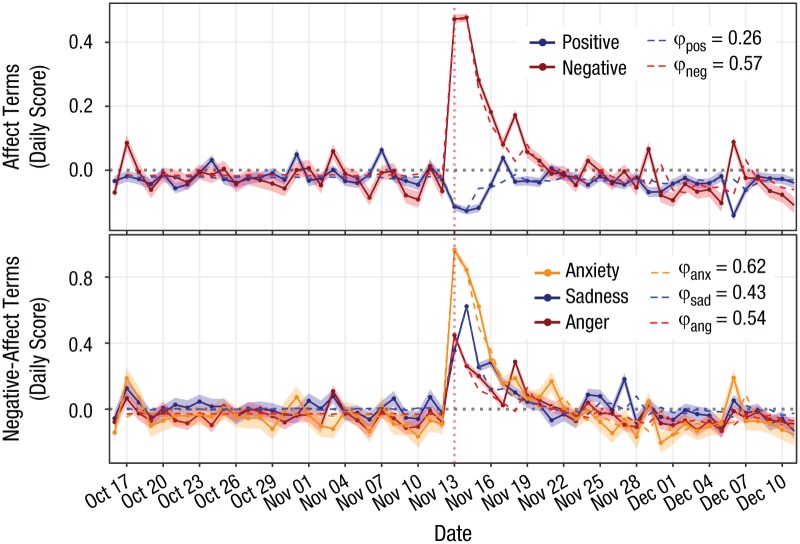
Daily scores of positive affect and negative affect (top) and anxiety, sadness, and anger (bottom). Points show median estimates over 10,000 bootstrapped samples, and shaded areas show their 95% confidence intervals. Dashed lines show model fits, along with the estimated values of each ϕ. The dotted vertical line marks the day of the attacks.

Figure 2 shows the daily normalized scores of emotion terms over a period of 8 weeks centered on the attacks. There is a strong response toward more frequent negative affect and a weaker response toward a lower frequency of positive affect. There is evidence of a collective negative emotion with significant memory in negative affect (ϕ = 0.57, 95% CI = [0.418, 0.716], *p* < .001), as hypothesized. The time series of positive affect shows some signs of collective behavior (ϕ = 0.26, 95% CI = [0.042, 0.474], *p* < .05), but with a weaker memory than negative affect. These results indicate the existence of a collective emotion that kept the levels of negative affect above their baseline for more than a week and led to the positive values of ϕ for negative affect. This contrasts with individual emotional responses, which relax much faster and, in the absence of emotional feedback loops, would produce values of ϕ that are indistinguishable from zero.

The composition of the expression of negative affect can be seen in the bottom panel of Figure 2. The strongest response was of anxiety, followed by sadness starting from the day after the attacks. Surprisingly for a reaction to a terrorist attack, the weakest response was of anger. All three negative emotions show collective behavior (anxiety: ϕ = 0.62, 95% CI = [0.480, 0.766], *p* < .001; sadness: ϕ = 0.43, 95% CI = [0.272, 0.594], *p* < .001; anger: ϕ = 0.54, 95% CI = [0.31, 0.77], *p* < .001), in line with our observations for the negative-affect indicator.

**Fig. 3.**
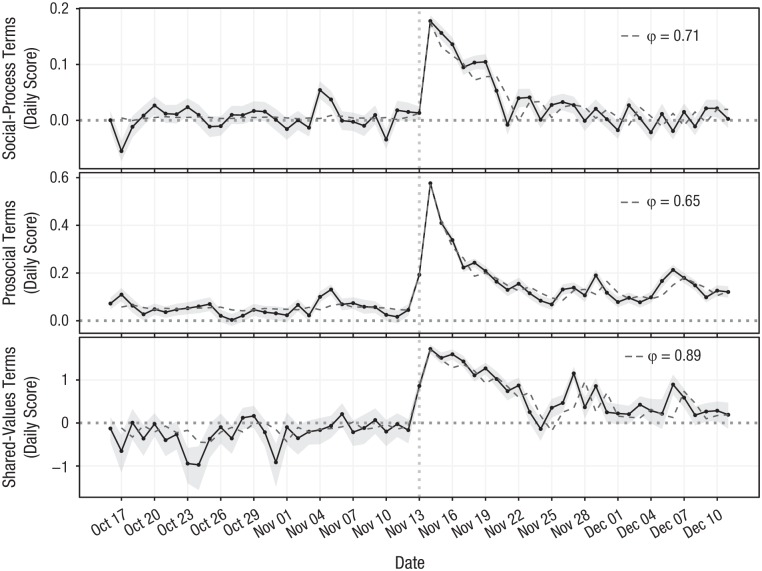
Daily scores of lexical indicators related to solidarity around the terrorist attacks: social-process terms (top), prosocial terms (middle), and shared-values terms (bottom). Dashed lines show model fits, and shaded areas show 95% bootstrapped confidence intervals of empirical data. The dotted vertical line marks the day of the attacks.

We repeated the same analysis for three lexical indicators related to solidarity: social-process terms, prosocial-behavior terms, and shared-values terms. Figure 3 shows the daily scores of these lexical indicators along with the model fits and the estimate of ϕ for each of them. They all display a similar pattern: Their spike is strong and is not exactly on the day of the attacks but the day afterward. They all have clear signs of collective behavior with significant memory (social process: ϕ = 0.71, 95% CI = [0.567, 0.86], *p* < .001; prosocial behavior: ϕ = 0.65, 95% CI = [0.566, 0.742], *p* < .001; shared values: ϕ = 0.89, 95% CI = [0.698, 1.09], *p* < .001). This suggests that solidarity follows collective emotions: All three indicators peaked 1 day later and show a memory pattern with a slow relaxation toward their baseline.

There is an interplay between social-process terms and affective terms that can be observed at faster time scales, in particular when testing the correlation between the content of a tweet and the content of the tweet written next by the same user shortly after the attacks. We fitted mixed-effects logistic regression models with a random intercept for each user in the sample, modeling the binary variable of a tweet containing a certain lexical indicator (e.g., social processes) as a function of the previous tweet containing another lexical indicator (e.g., positive affect) with a correction for autocorrelation and its interaction. We found that a tweet containing affective terms was predictive of the next tweet containing social-process terms (positive affect: β = 0.06, 95% CI = [0.04, 0.07], *p* < .001; negative affect: β = 0.08, 95% CI = [0.07, 0.10], *p* < .001). Similarly, we found that tweets containing social-process terms were also predictive of the following tweets containing affective terms, but with effects of smaller magnitude (both with β = 0.03, 95% CI = [0.02, 0.04], *p* < .001). More details about these results are presented in the Supplemental Material, including a comparison of magnitudes and interaction effects.

### Individual-level analysis

To test the individual effects of collective effervescence suggested by Durkheim’s theory, we performed an individual-level analysis linking short-term emotional expression after the attacks with long-term expressions related to solidarity and affect. We identified individuals showing emotional synchronization as those using affect terms (both positive and negative) more frequently during the 2 weeks after the attacks than in their individual baseline during the 3 months before the attacks. In this way, we divided individuals into a high-emotional-synchronization group and a low-emotional-synchronization group.

**Fig. 4.**
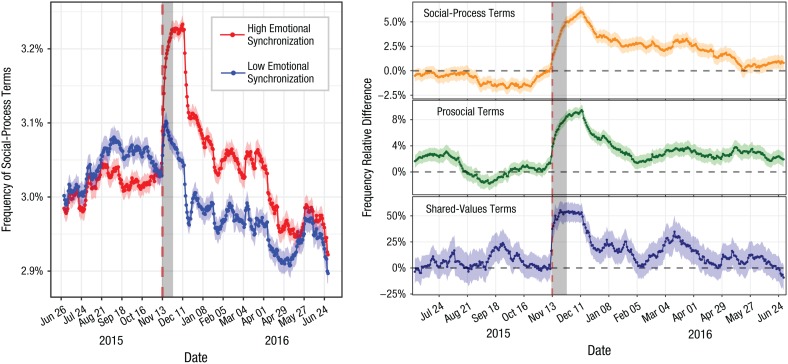
Frequency of time-series social-process terms for individuals in the high- and low-emotional-synchronization groups (left) and time series of the relative frequency difference between groups for social-process terms, prosocial terms, and shared-values terms (right). Both panels show values averaged over sliding windows of 1 month. The gray area marks the 2 weeks used to measure emotional synchronization, and shaded areas around solid lines show 95% bootstrapped confidence intervals. The dashed vertical lines mark the day of the attacks.

The left panel of Figure 4 shows the time series of the frequency of social-process terms over a long period before and after the attacks, differentiating the frequency between emotional-synchronization groups. Before the attacks, both groups used a similar frequency of social-process terms, but after the attacks, individuals in the high-emotional-synchronization group (i.e., who took part in the collective emotion) used more social-process terms on average than individuals in the low-emotional-synchronization group. This difference was persistent over time, with a clear distance even months after the attacks took place.

The same phenomenon can be observed for terms related to prosocial behavior and for terms about shared values. The right panel of Figure 4 shows the difference between the high-emotional-synchronization group and the low-emotional-synchronization group for each of the three lexical indicators. It can be observed that, in all three cases, there was a significant positive difference toward the high-emotional-synchronization group that lasted for months after the attacks, with shared values going back to the baseline the fastest.

Whereas the results described above illustrate the effect of collective emotions in long-term lexical indicators related to solidarity, it is necessary to test the robustness of these observations to self-selection confounds and covariates that can be measured in user-level data. We verified these results in mediation analyses in which the treatment was the average frequency of emotional terms tweeted by a user in the 2 weeks after the attacks after correction for self-selection through personality-related lexical indicators. The outcome variable of these analyses was the long-term frequency of terms of a certain kind (e.g., social-process terms), and the mediator variable was the short-term frequency of those terms in the 2 weeks after the attacks. We used vectors of activity and personality-related lexical indicators as covariates to ensure a robust correction for these measurable confounds. We rescaled all indicators before analysis to have comparable estimates.

We found a significant positive direct effect of corrected emotion levels on long-term use of social-process terms (average direct effect, or ADE = 0.022, 95% CI = [0.011, 0.03], *p* < .001), which was partially mediated by the short-term use of social-process terms (average causal mediation effect, or ACME = 0.016, 95% CI = [0.013, 0.02], *p* < .001). Results were similar for prosocial terms, with a significant positive direct effect of corrected emotion levels (ADE = 0.0157, 95% CI = [0.0043, 0.03], *p* < .01) and a positive effect mediated on the short-term use of prosocial terms (ACME = 0.0092, 95% CI = [0.0072, 0.01], *p* < .001). For shared values, we found no significant direct effect of corrected emotion levels (*p* = .27), but there was a significant effect mediated on the short-term level of shared values (ACME = 0.0034, 95% CI = [0.0004, 0.01], *p* < .001). We explored further the long-term effects on the levels of use of positive-affect and negative-affect terms. We found a significant positive direct effect of corrected emotion levels on long-term expression of positive affect (ADE = 0.0678, 95% CI = [0.0499, 0.09], *p* < .001), which was partially mediated by the short-term expression of positive affect (ACME = 0.0567, 95% CI = [0.0421, 0.07], *p* < .001). On the contrary, for negative affect, we found no significant direct effects (*p* = 0.42), and we found a positive effect only when it was mediated by the short-term expression of negative affect (ACME = 0.0563, 95% CI = [0.0477, 0.07], *p* < .001).

Despite these long-term effects on solidarity indicators and affect, individuals with high short-term emotionality after the attacks did not reference the attacks more frequently in the long run. A negative binomial model with zero inflation shows that users with high emotionality after the attacks used terms related to the attacks less frequently in the 3-month period (for more details, see the Supplemental Material). This suggests that the effects on solidarity and affect were not generated by later discussions about the attacks.

## Discussion

We analyzed collective emotional synchronization in a naturalistic, observational study of behavior in vivo as manifested in social media. Although our approach was very close to social reality, one should keep in mind that our analysis was purely observational and thus has limitations to fully reveal causal mechanisms. Users self-selected into the collective emotion by posting emotional tweets after the attacks, which is far from a situation in which we have control over who is involved in the collective emotion. We corrected for personality-related psycholinguistic features of the users to try to get closer to a random treatment, but we must still warn about the possible role of behavioral traits in our analysis. In addition, we could analyze only individuals who were active on Twitter and shared a location that we could map to France. This kind of geographic sample has been shown to be more consistent than keyword-based samples (King et al., 2017; Zhang, Hill, & Rothschild, 2018), but we still do not know if there are behavioral or emotional differences between the users in this kind of sample and the population in general. Only a combination of methods can prevent future research from falling into the trap of big-data hubris (Lazer, Kennedy, King, & Vespignani, 2014) of assuming that a data set is free of limitations just because it is large. Future investigations can combine large-scale observational analyses with established and representative survey methods, as recently shown for the case of a campus lockdown (Jones, Thompson, Dunkel Schetter, & Silver, 2017).

Our results shed light on a case of the response to a collective trauma, which we interpreted as an instance of social resilience through the lens of Durkheim’s theory. We must note that we measured only three responses associated with different aspects of solidarity (social processes, prosocial terms, and shared values) and not the function of social resilience itself. Future studies in more than one collective trauma can elucidate the process of social resilience and potentially reveal the dynamics of how societies recover after disaster hits. We found that long-term expressions of solidarity are related to the previous participation in collective emotions, but we also found effects for long-term expressions of affect and correlations in both directions in shorter time scales. This shows that the process we observed is not a simple cause and effect between collective emotions and solidarity. The most likely scenario is an interplay at various time scales, in which both solidarity and emotions influence each other.

Our observations of collective responses show that sadness peaks 1 day after the attacks, whereas anger and anxiety peak on the day of the attacks. This could be explained by the lower arousal levels of sadness, which would delay expression in tweets, or by the expression of sadness being secondhand, that is, the sadness of people not directly affected by the attacks but expressed in relation to those who were harmed.

Our work touched only the tip of the iceberg of the phenomena captured by Durkheim’s theory. Durkheim’s principle of collective effervescence can also be observed in the timing patterns of large-scale social media activity (González-Bailón, 2013). Furthermore, collective emotions are of relevance in further psychological phenomena, such as group-based emotions (Goldenberg, Saguy, & Halperin, 2014) and collective action (Alvarez et al., 2015). In addition, we still do not know the precise conditions under which collective emotions lead to broad solidarity or are also accompanied by intolerance toward out-group members. We noticed weaker effects of emotions in shared values than in social processes and in prosocial language, which calls for a theoretical approach that models the similarities and differences between these behaviors and phenomena.

Our results shed new light on the social function of collective emotions, illustrating that a society hit by a collective trauma does not just respond with simultaneous negative emotions. The collective experience of emotions leads to long-term solidarity in the activation of social processes, in the use of language related to prosocial behavior, and in the expression of positive affect. These findings suggest that it is not despite our distress that we are more united after a terrorist attack, but it is precisely because of our shared distress that our bonds become stronger and our society adapts to face the next threat.

## Supplemental Material

GarciaOpenPracticesDisclosure – Supplemental material for Collective Emotions and Social Resilience in the Digital Traces After a Terrorist Attack Click here for additional data file. Supplemental material, GarciaOpenPracticesDisclosure for Collective Emotions and Social Resilience in the Digital Traces After a Terrorist Attack by David Garcia and Bernard Rimé in Psychological Science

## Supplemental Material

GarciaSupplementalMaterial – Supplemental material for Collective Emotions and Social Resilience in the Digital Traces After a Terrorist Attack Click here for additional data file. Supplemental material, GarciaSupplementalMaterial for Collective Emotions and Social Resilience in the Digital Traces After a Terrorist Attack by David Garcia and Bernard Rimé in Psychological Science

## Notes

1. An online interactive visualization of the results presented in this article can be viewed at http://dgarcia.eu/ParisAttacks.html.
