# Supplementary material for: Collective Emotions and Social Resilience in the Digital Traces After a Terrorist Attack
Source: Psychol Sci. 2019 Mar 13;30(4):617–28. doi: 10.1177/0956797619831964 (PMC6472144; doi:10.1177/0956797619831964)
Supplement: GarciaSupplementalMaterial – Supplemental material for Collective Emotions and Social Resilience in the Digital Traces After a Terrorist Attack [file GarciaSupplementalMaterial.pdf]

Collective emotions and social resilience in the digital traces after a terrorist attack - Supplementary Materials

David Garcia[1,2] and Bernard Rimé[3]

[1]Section for Science of Complex Systems, CeMSIIS,

Medical University of Vienna, Vienna, Austria

[2]Complexity Science Hub Vienna, Vienna, Austria

[3]Psychological Sciences Research Institute,

Université de Louvain at Louvain-la-Neuve, Belgium

Collective emotions and social resilience in the digital traces after a terrorist attack - Supplementary Materials

Twitter data

For a month after the attacks, we used the Twitter search API on a daily basis to retrieve tweets that contained any of the hashtags included in Table 1. We removed all tweets that were not in French, as specified by Twitter's metadata and language detection software (Shuyo, 2010). Out of a total of 492,994 users found in that tweet sample, 287,996 (58%) of them had a non-empty user location field on their profile, which we used to select users that disclose living in France. More precisely, we selected users through the following criteria:

1. Produced less than 3,200 tweets in our observation period
2. Had a ratio of followers to friends between 0.1 and 10
3. Provided a nonempty location field in their Twitter profile
4. Their location field text could be mapped to a European France location through Google Maps Geocode API

The first two rules exclude bots (high amount of friends compared to followers or high activity) and mass media or corporate accounts (high amount of followers compared to friends or high activity). Rules 3 and 4 ensure that all users have self-disclosed their location that places them in France. This geocoding step of user profiles differs from the technique of geolocation by tweet GPS coordinates, as we do not require the users to actively disclose their location in the metadata of their tweets. This leaves a user sample to 62,114 user accounts in the country affected by the attacks. Some of these users reported their locations in a general way (e.g. "France") that did not lead to a precise location estimate (longitude and latitude) in

the geocoding step, but many had more precise locations that allowed us to have an insight into the spatial distribution of the data, shown on the map of Figure 1 of the Main Text.

#parisattacks	#PrayForParis	#Paris	#PorteOuverte
#JeSuisParis	#NousSommesUnis	#PeaceForParis	#PrayForFrance
#jesuisparisian	#FranceUnderAttack	#PrayForTheWorld	#NotInMyName
#MuslimsAreNotTerrorist	#ViveLaFrance	#notafraid	#Bataclan
#Attacks	#attaque	#daesh	#radical
#terror	#terreur	#molenbeek	

Table 1

Hashtags used to sample users after the terrorist attacks of November 13th.

In early 2017, we retrieved the timeline of tweets of the users in our sample, removing all retweets and all tweets not detected to be in French. We limit our analysis to the period between April 1st 2015 and June 30th 2016, including 17,899,591 original tweets in French. Some users in our sample (447 users, less than 1%) had only produced retweets and tweets not in French during that period, and thus are excluded from our subsequent analyses.

Ethical considerations

All data used in this article is publicly available archival data that was produced and posted by the self-selected users. No private information of any user was retrieved at any point. The study does not include any manipulation or intervention and constituted a purely observational study. In line with the growing consensus in ethics (Metcalf & Crawford, 2016), our work is excluded from ethics review. Minimum risk is associated with our research and the inclusion through hashtags constitutes sufficiently informed consent and respects user expectations. Before carrying this study, we assessed its potential to shed light in important social and emotional processes, bringing opportunities to understand social resilience and the impact of terrorism. These benefits greatly outweigh the risks of analyzing this kind of public archival data, in particular given the self-selection of users and the observational nature of this study. The research presented in here was considered exempt from ethics review by the Ethics Commission of the Medical University of Vienna (decision 1600/2018).

Validating French LIWC dictionaries for Tweets

We validated the emotion classes of LIWC in French against a dataset with manually annotated tweets from the DEFT 2015 competition (Fraisse, Grouin, Hamon, Paroubek, & Zweigenbaum, 2015). Among various tasks, this competition included a sentiment analysis task in which participants classified tweets as positive (+), negative (-), or neutral (0). It als included an emotion detection task to classify emotions from tweet text. Among the emotion classes, it included “Colere”, which literally translates to “anger” in English, “Tristesse”, literally translated as “sadness”, and “Peur”, which literally means “fear” but in the coding instructions it included “anxiety” as an example (see `https://deft.limsi.fr/2015/descriptionTaches.fr.php?lang=fr`). For our validation exercise, we merge training and test datasets of the task, since the LIWC method is unsupervised and can be validated against the whole dataset. The organizers of DEFT could only share the ids of tweets, which need to be looked up through the Twitter API to recover their text. This way we reconstructed 10,250 tweets for the first task and 4,123 tweets for the second task.

To test the validity of the French adaptation of LIWC when applied to tweets, we measured the frequencies of PA, NA, anger, sadness, and anxiety LIWC terms in each tweet of the dataset. The results are shown on Table 2, evidencing significant and sizable differences in the frequency of each term class when comparing tweets with the corresponding annotation versus the rest of the dataset. This is specially notable for the negative affect classes, as for example the text of tweets annotated as negative contain 5.2 times the amount of negative terms of the text of tweets annotated as neutral or positive.

We also evaluate the French translation of the prosocial terms dictionary of Frimer, Schaefer, and Oakes (2014) in Twitter. We selected a set of seven French Twitter accounts that aim to promote prosocial behavior (MSF_france, SOSMedFrance, MdM_France,

	PA (not +)	PA (+)	Ratio	χ^2	p-value	+ count	total
+	1.9%	3.99%	2.1	676.49	$< 10^{-10}$	3,243	10,250
	NA (not -)	NA (-)	Ratio	χ^2	p-value	- count	total
-	0.64%	3.29%	5.2	1830.1	$< 10^{-10}$	2,387	10,250
	anger (not Col.)	anger (Col.)	Ratio	χ^2	p-value	Col. count	total
Colere	0.53%	2.44%	4.6	245.77	$< 10^{-10}$	252	4,123
	sadness (not Tri.)	sadness (Tri.)	Ratio	χ^2	p-value	Tri. count	total
Tristesse	0.37%	3.86%	10.33	234.16	$< 10^{-10}$	46	4,123
	anxiety (not Peur)	anxiety (Peur)	Ratio	χ^2	p-value	Peur count	total
Peur	0.17%	2.17%	12.5	657.19	$< 10^{-10}$	349	4,123

Table 2

LIWC emotion validation results against annotations reconstructed from Fraisse et al. (2015).

UNICEF_france, restosducoeur, CroixRouge, caritasfrance) and retrieved their timelines up to the last 3200 tweets in March 2017, gathering a total of 22,392 tweets. We applied the prosocial terms dictionary and compared the frequencies against the 6 month baseline of tweets in our dataset. Prosocial term frequency in prosocial accounts is 3.35%, while in the baseline dataset is 1.31% ($\chi^2 = 14357$, p-value $< 10^{-10}$). This means that prosocial terms have a frequency ratio of 2.56 compared to the text of tweets in the baseline dataset, a significant and sizable difference that evidences the validity of the lexicon and its French translation.

Analysis of collective behavior

For each lexical indicator we calculated its daily mean $X(t)$, and then we computed a normalized score $Z_X(t) = log(X(t)/X_b)$, where X_b is the baseline value of the corresponding weekday. This logarithmic transformation reduces the skewness of the ratio between term class frequencies, such that $Z_X(t)$ lies in the interval $[-\infty, +\infty]$ instead of in the interval $[0, +\infty]$. A negative score indicates that the daily frequency is below the baseline and a positive score that the daily frequency is above the baseline.

We fitted the temporal evolution of each normalized score $Z_X(t)$ of each linguistic variable X through a time series model of the form:

$$Z_X(t) = \varphi * Z_X(t-1) * \Theta[t > t_1] + \varphi_{pre} * Z_X(t-1) * \Theta[t < t_1]$$
$$+D_1 * \Theta[t = t_1] + D_2 * \Theta[t = t_2] + c + \epsilon$$

In the above equation, c is a time-independent intercept. D_1 and D_2 measure the size of the shock due to the attacks happening during the night of t_1 (Nov 13th) and being widely reported during day t_2 (Nov 14th). The parameter φ quantifies the memory of the time series after the attacks as the linear relationship between $Z_X(t-1)$ and $Z_X(t)$. Positive values of φ can be generated by synchronized behavior, as explained by the agent-based model described below. The parameter φ_{pre} measures the memory of $Z_X(t)$ before the attacks, which should be much smaller than φ and very close to zero if the attacks triggered emotion sharing feedback loops that were not active before.

We fitted models with the `bayesglm` function of the `arm` R package (Gelman, Jakulin, Pittau, & Su, 2008), taking weakly informative priors for all parameters. We report the median value and the 95% CI of the posterior distribution of each parameter, along with the p-values of standard statistical tests. After fitting, we ran regression diagnostics to validate the assumptions of the above model.

To shed light on the relationship between synchronization of behavior and the statistical fit of the above model, we designed an agent-based model of the reaction of a community to a collective trauma. We combined computational models of collective emotions (Schweitzer & Garcia, 2010) with previous empirical results in experiments and diary studies in the design, calibration, and analysis of this model. This way we aim to bridge the level of collective behavior with the dynamics of individuals through the modeling of how they synchronize their behavior (Smith & Conrey, 2007). We simulated a society composed of agents that have an observable variable $x(t)$ that represents a state such as affect or prosocial term use in a day.

Agent states are driven by three mechanisms:

1. Internal dynamics: a combination of random effects sampled from a normal distribution of zero mean with a relaxation tendency $\gamma = 0.005/min$ that drives state $x(t)$ towards its baseline $x = 0$. This internal dynamics are based on the empirical results of emotion dynamics through self-reports (Kuppens, Oravecz, & Tuerlinckx, 2010).

2. Reaction to the attack: a fixed impulse of size $D = 1$ that is active during the day of the attacks. This impulse is perceived by all agents at the same time and marks the onset of any possible collective behavior. This principle is based on previous models of collective emotions in online social media (Schweitzer & Garcia, 2010).

3. Synchronization of behavior: Agents react to each other at a rate α that is directly proportional to the absolute value of their internal state, $x(t)$. As a simplification, we sample the peer of this synchronization at random and add a delay in the reception of an online message that we sample from an exponential distribution of parameter $\lambda = 0.001$. After that time the agent changes its state to the state of the other agent, as an event of emotional or behavioral synchronization. This effect follows the results found in experiments of emotional discussions in online media (Garcia, Kappas, Küster, &

Schweitzer, 2016).

The above design allows us to calibrate the intensity of behavior synchronization through the parameter α. The relationship between this strength of synchronization and the internal relaxation tendency captured by γ will define the shape of the trend of the aggregate response, which we quantify in our empirical analysis through parameter φ.

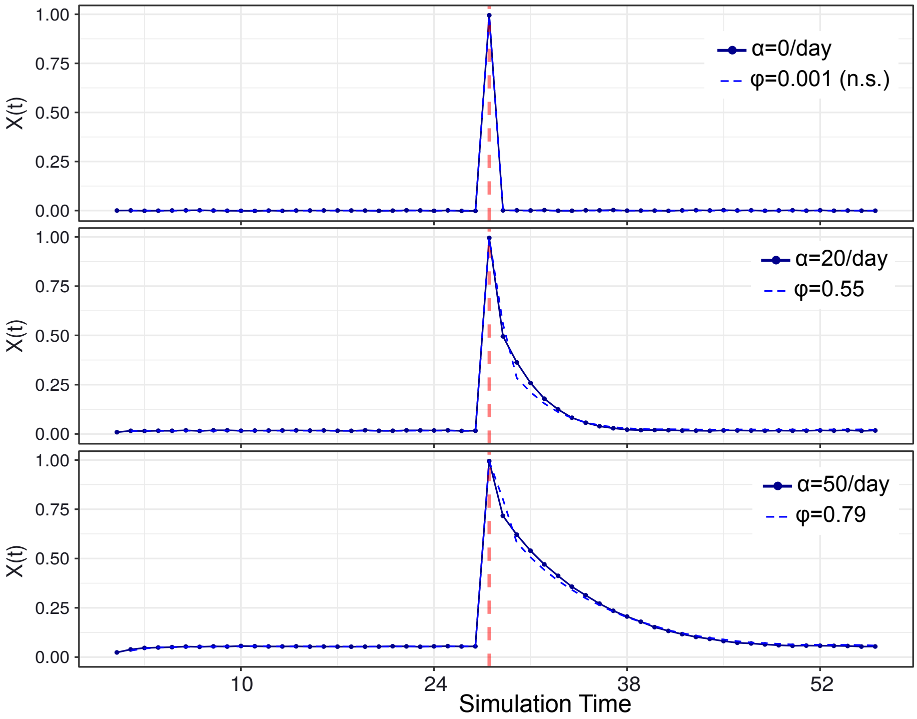

Figure 1. Results of three simulations of the model for a reaction with no synchronization (top), intermediate level of synchronization (middle), and strong synchronization (bottom).

Simulation examples can be found in Figure 1, which illustrate the relationship between α and φ. When $\alpha = 0$, there is no synchronization of behavior, and as a result, the collective average response $X(t)$ relaxes quickly towards zero and hence the value of φ extremely close to zero. Examples with α vales of 20/day and 50/day lead to collective responses with longer memory as quantified by significant estimates of $\varphi = 0.55$ and $\varphi = 0.79$. Furthermore,

regression on the simulation results lead to positive estimates of φ when α is high and a non-significant one for $\alpha = 0$, as reported in Table 3.

coefficient	$\alpha = 0/day$	$\alpha = 20/day$	$\alpha = 50/day$
Intercept	0.0001	$\mathbf{0.013}^{***}$	$\mathbf{0.017}^{***}$
φ (before shock)	0.217	0.216	$\mathbf{0.695}^{***}$
φ (after shock)	0.0009	$\mathbf{0.548}^{***}$	$\mathbf{0.789}^{***}$
D (shock size)	$\mathbf{0.995}^{***}$	$\mathbf{0.978}^{***}$	$\mathbf{0.94}^{***}$
R^2	0.999	0.989	0.994
N	55	55	55

$^{***}p < 0.001$, $^{**}p < 0.01$, $^{*}p < 0.05$

Table 3

Regression results of the memory model for a simulation collective responses. Increases in the synchronization parameter α lead to collective responses with φ values significantly above zero. This shows the relationship between the agent synchronization and the memory pattern of the collective response $X(t)$.

Time series model fits

coefficient	PA		NA	
Intercept	$\mathbf{-0.024}^{***}$	$[-0.034, -0.014]$	$\mathbf{-0.018}^{*}$	$[-0.032, -0.003]$
φ_{pre}	-0.07	$[-0.316, 0.181]$	-0.065	$[-0.475, 0.34]$
φ	$\mathbf{0.26}^{*}$	$[0.042, 0.474]$	$\mathbf{0.57}^{***}$	$[0.418, 0.716]$
D_1	$\mathbf{-0.09}^{**}$	$[-0.148, -0.028]$	$\mathbf{0.48}^{***}$	$[0.38, 0.58]$
D_2	$\mathbf{-0.07}^{*}$	$[-0.132, -0.008]$	$\mathbf{0.22}^{***}$	$[0.104, 0.344]$
R^2	0.322		0.814	
N	56		56	

$^{***}p < 0.001$, $^{**}p < 0.01$, $^{*}p < 0.05$

Table 4

Regression results of the memory model for affective terms.

The fit results of the PA and NA models are reported on Table 4. Both models have residual distributions roughly normally distributed (Shapiro-Wilk statistics of 0.88 and 0.91 respectively). KPSS tests cannot reject the null hypothesis that residuals are stationary ($p > 0.1$ in both cases). In addition, the square root of the absolute value of residuals are not significantly correlated with fitted values ($p = 0.23$ for PA, $p = 0.08$ for NA).

coefficient	Sadness		Anger		Anxiety	
Intercept	0.0003	$[-0.018, 0.019]$	$-\mathbf{0.027}^{*}$	$[-0.047, -0.007]$	$-\mathbf{0.031}^{*}$	$[-0.058, -0.01]$
φ_{pre}	-0.12	$[-0.57, 0.35]$	0.043	$[-0.33, 0.414]$	0.11	$[-0.283, 0.53]$
φ	$\mathbf{0.43}^{***}$	$[0.272, 0.594]$	$\mathbf{0.54}^{***}$	$[0.31, 0.77]$	$\mathbf{0.62}^{***}$	$[0.480, 0.766]$
D_1	$\mathbf{0.344}^{***}$	$[0.213, 0.478]$	$\mathbf{0.47}^{***}$	$[0.336, 0.607]$	$\mathbf{0.99}^{***}$	$[0.82, 1.18]$
D_2	$\mathbf{0.46}^{***}$	$[0.32, 0.60]$	0.042	$[-0.124, 0.203]$	$\mathbf{0.27}^{*}$	$[0.051, 0.49]$
R^2	0.725		0.63		0.846	
N	56		56		56	

$^{***}p < 0.001$, $^{**}p < 0.01$, $^{*}p < 0.05$

Table 5

Regression results of the memory model for negative affect classes.

The fit results of the three negative emotion models are reported on Table 5. All models have residual distributions roughly normally distributed (Shapiro-Wilk statistics of 0.97, 0.86, and 0.94 respectively). KPSS tests cannot reject the null hypothesis that residuals are stationary ($p > 0.1$ in all cases). In addition, the square root of the absolute value of residuals are not significantly correlated with fitted values ($p = 0.14$ for sadness, $p = 0.15$ for anger, and $p = 0.34$ for anxiety).

coefficient	Social Processes		Prosocial Terms		Shared Values	
Intercept	0.004	$[-0.003, 0.011]$	$\mathbf{0.041}^{***}$	$[0.025, 0.057]$	-0.063	$[-0.2, 0.067]$
φ_{pre}	0.08	$[-0.20, 0.365]$	0.23	$[-0.07, 0.529]$	$\mathbf{0.403}^{*}$	$[0.011, 0.79]$
φ	$\mathbf{0.712}^{***}$	$[0.567, 0.86]$	$\mathbf{0.65}^{***}$	$[0.566, 0.742]$	$\mathbf{0.89}^{***}$	$[0.698, 1.09]$
D_1	0.007	$[-0.04, 0.055]$	$\mathbf{0.141}^{***}$	$[0.077, 0.205]$	$\mathbf{0.97}^{**}$	$[0.29, 1.65]$
D_2	$\mathbf{0.162}^{***}$	$[0.114, 0.207]$	$\mathbf{0.407}^{***}$	$[0.342, 0.471]$	$\mathbf{1.00}^{**}$	$[0.315, 1.67]$
R^2	0.727		0.906		0.715	
N	56		56		56	

$^{***}p < 0.001$, $^{**}p < 0.01$, $^{*}p < 0.05$

Table 6

Regression results of the memory model for terms related to social resilience.

The fit results of the three social resilience terms models are reported on Table 6. All models have residual distributions roughly normally distributed (Shapiro-Wilk statistics of 0.92, 0.93, and 0.98 respectively). KPSS tests cannot reject the null hypothesis that residuals are stationary ($p > 0.1$ in all cases but for shared values, where a weak autocorrelation is present). In addition, the square root of the absolute value of residuals are not significantly correlated with fitted values ($p = 0.78$ for social processes, $p = 0.63$ for prosocial terms, and $p = 0.22$ for shared values).

Individual-level analysis

Correction for self-selection

Term	Estimate	95% CI
Intercept	$\mathbf{0.0365}^{***}$	$[0.0355, 0.0375]$
Positive Affect	$\mathbf{0.3095}^{***}$	$[0.2942, 0.3248]$
Negative Affect	$\mathbf{0.3350}^{***}$	$[0.3100, 0.3600]$
Social Processes	-0.0098	$[-0.0303, 0.0108]$
First Person Singular	$\mathbf{0.2412}^{***}$	$[0.2262, 0.2562]$
$log(N_{tweets})$	$-\mathbf{0.0015}^{***}$	$[-0.0018, -0.0012]$
R^2	0.093	
N	49001	

$^{***}p < 0.001$, $^{**}p < 0.01$, $^{*}p < 0.05$

Table 7

Self-selection model based on personality correlates measured during the baseline period.

Table 7 presents the self-selection model of participating in the collective emotion as a function of personality-related lexical indicators and activity levels. Users with high emotionality in the two weeks after the attacks also had high emotionality in the three months before, higher use of first person singular terms, and slightly lower activity levels.

Tweet-level analysis

	$Soc_{PA}(t+1)$	$PA_{Soc}(t+1)$	$Soc_{NA}(t+1)$	$NA_{Soc}(t+1)$
intercept	$\mathbf{-0.76}^{***}$	$\mathbf{-0.63}^{***}$	$\mathbf{-0.76}^{***}$	$\mathbf{-1.21}^{***}$
95% CI	$[-0.77, -0.75]$	$[-0.64, -0.62]$	$[-0.77, -0.75]$	$[-1.22, -1.20]$
$Soc(t)$	$\mathbf{0.20}^{***}$	$\mathbf{0.03}^{***}$	$\mathbf{0.22}^{***}$	$\mathbf{0.03}^{***}$
95% CI	$[0.18, 0.21]$	$[0.02, 0.04]$	$[0.21, 0.23]$	$[0.02, 0.04]$
$PA(t)$	$\mathbf{0.06}^{***}$	$\mathbf{0.15}^{***}$		
95% CI	$[0.04, 0.07]$	$[0.13, 0.16]$		
$Soc(t) * PA(t)$	-0.01	$\mathbf{0.04}^{***}$		
95% CI	$[-0.03, 0.008]$	$[0.02, 0.06]$		
$NA(t)$			$\mathbf{0.08}^{***}$	$\mathbf{0.21}^{***}$
95% CI			$[0.07, 0.10]$	$[0.20, 0.23]$
$Soc(t) * NA(t)$			$\mathbf{-0.06}^{***}$	0.01
95% CI			$[-0.09, -0.04]$	$[-0.01, 0.04]$
AIC	1130448	1150383	1130418	996823
N	890994	890994	890994	890994
N users	53004	53004	53004	53004
Var(user)	0.23	0.23	0.23	0.24

$^{***}p < 0.001$, $^{**}p < 0.01$, $^{*}p < 0.05$

Table 8

Results of mixed models for tweet-level analysis.

Table 8 presents an overview of the tweet-level models using the data for the two weeks after the attacks. All models are mixed-effects logistic regression models with a dependent variable that indicates if the next tweet of a user contains at least one term of a lexical indicator. Both affect and social process terms are positively associated with each other, but the association from affect to social process terms is stronger, as Wald 95% confidence intervals of the corresponding parameters do not overlap. Reporting these intervals with more precision, the effect of PA on social processes lies on the interval $[0.0449, 0.0698]$, while the effect of social processes on PA is on the interval $[0.0162, 0.0418]$. Since intervals do not overlap and the latter one is below, we can conclude that the relationship is stronger in the direction from PA to social processes. A similar pattern can be observed for NA: the interval of the effect of social processes on NA ($[0.0189, 0.0439]$) is below the one of NA on social processes ($[0.0685, 0.0953]$). In this case, we must note that there is an interaction effect in this last model that attenuates the effect of NA when there is also a social process term in the previous tweet, i.e. that NA terms predict changes from using no social process terms in a tweet to using at least one in the following tweet.

Individual effects models

Effect	Social	Prosocial	Shared values	PA	NA
ACME	$\mathbf{0.016}^{***}$	$\mathbf{0.0092}^{***}$	$\mathbf{0.0034}^{***}$	$\mathbf{0.0567}^{***}$	$\mathbf{0.0563}^{***}$
95% CI	$[0.013, 0.02]$	$[0.0072, 0.01]$	$[0.0004, 0.01]$	$[0.0421, 0.07]$	$[0.0477, 0.07]$
ADE	$\mathbf{0.022}^{***}$	$\mathbf{0.0157}^{**}$	-0.0053	$\mathbf{0.0678}^{***}$	0.0052
95% CI	$[0.011, 0.03]$	$[0.0043, 0.03]$	$[-0.0156, 0.00]$	$[0.0499, 0.09]$	$[-0.0074, 0.02]$
Total Effect	$\mathbf{0.038}^{***}$	$\mathbf{0.0249}^{***}$	-0.0019	$\mathbf{0.1245}^{***}$	$\mathbf{0.0615}^{***}$
95% CI	$[0.027, 0.05]$	$[0.0136, 0.04]$	$[-0.0108, 0.01]$	$[0.1122, 0.14]$	$[0.0504, 0.07]$
Prop. Med.	$\mathbf{0.417}^{***}$	$\mathbf{0.3698}^{***}$	-1.85	$\mathbf{0.4556}^{***}$	$\mathbf{0.9152}^{***}$
95% CI	$[0.297, 0.61]$	$[0.2351, 0.69]$	$[-10.5, 12.01]$	$[0.3364, 0.58]$	$[0.7394, 1.14]$

$^{***}p < 0.001$, $^{**}p < 0.01$, $^{*}p < 0.05$

Table 9

Mediation analysis results

Table 9 shows the results of mediation analysis using the `mediation` R package (Imai, Keele, Tingley, & Yamamoto, 2010),

Long-term attack references

Term	Estimate	95% CI
Count model: Intercept	$\mathbf{0.3306}^{***}$	$[0.3033, 0.3579]$
Count model: E_c	$-\mathbf{4.5462}^{***}$	$[-5.0645, -4.0278]$
Count model: N_{tweets}	$\mathbf{0.0071}^{***}$	$[0.0069, 0.0073]$
Zero model: Intercept	$\mathbf{1.2527}^{***}$	$[1.1426, 1.3627]$
Zero model: E_c	-0.5051	$[-1.8141, 0.8038]$
Zero model: N_{tweets}	$-\mathbf{0.1663}^{***}$	$[-0.1813, -0.1513]$
AIC	173139.3889	
Log Likelihood	-86562.6945	
Num. obs.	49001	

$^{***}p < 0.001$, $^{**}p < 0.01$, $^{*}p < 0.05$

Table 10

Regression results of the negative binomial model with zero inflation of the count attack references in the three-month period. The model had a significant estimate of $log(\Theta)$ of -0.7297.

Table 10 shows the results of a zero-inflated negative binomial model fitted with the `zeroinfl` function of the `pscl` R package (Zeileis, Kleiber, & Jackman, 2008). Beyond those results, the fraction of tweets with a reference to the attacks over all tweets of each user in the three-month period is negatively correlated with affect and social process terms in the same period. The correlation with PA is -0.1273 (CI=$[-0.1360, -0.1186]$, $p < 10^{-6}$), with NA is -0.1559 (CI=$[-0.1645, -0.1473]$, $p < 10^{-6}$), and with social process terms is -0.0706 (CI=$[-0.0794, -0.0618]$, $p < 10^{-6}$)

References

Fraisse, A., Grouin, C., Hamon, T., Paroubek, P., & Zweigenbaum, P. (2015). *Deft 2015 eleventh french text mining challenge.* Retrieved from `https://deft.limsi.fr/2015/index.en.php?lang=en`

Frimer, J. A., Schaefer, N. K., & Oakes, H. (2014). Moral actor, selfish agent. *Journal of Personality and Social Psychology*, *106*(5), 790–802. doi: 10.1037/a0036040

Garcia, D., Kappas, A., Küster, D., & Schweitzer, F. (2016). The dynamics of emotions in online interaction. *Royal Society Open Science*, *3*(8), 160059. doi: 10.1098/rsos.160059

Gelman, A., Jakulin, A., Pittau, M. G., & Su, Y.-S. (2008). A weakly informative default prior distribution for logistic and other regression models. *The Annals of Applied Statistics*, 1360–1383. doi: 10.1214/08-AOAS191

Imai, K., Keele, L., Tingley, D., & Yamamoto, T. (2010). Causal mediation analysis using r. In H. D. Vinod (Ed.), *Advances in social science research using r* (pp. 129–154). New York: Springer-Verlag. doi: 10.1007/978-1-4419-1764-5_8

Kuppens, P., Oravecz, Z., & Tuerlinckx, F. (2010). Feelings change: accounting for individual differences in the temporal dynamics of affect. *Journal of Personality and Social Psychology*, *99*(6), 1042–1060. doi: 10.1037/a0020962

Metcalf, J., & Crawford, K. (2016). Where are human subjects in big data research? the emerging ethics divide. *Big Data & Society*, *3*(1), 2053951716650211. doi: 10.1177/2053951716650211

Schweitzer, F., & Garcia, D. (2010). An agent-based model of collective emotions in online communities. *The European Physical Journal B-Condensed Matter and Complex Systems*, *77*(4), 533–545. doi: 10.1140/epjb/e2010-00292-1

Shuyo, N. (2010, July). *Language detection library for java* (Vol. 7). Retrieved from `https://github.com/shuyo/language-detection`

Smith, E. R., & Conrey, F. R. (2007). Agent-based modeling: A new approach for theory building in social psychology. *Personality and Social Psychology Review*, *11*(1), 87–104. doi: 10.1177/1088868306294789

Zeileis, A., Kleiber, C., & Jackman, S. (2008). Regression models for count data in R. *Journal of Statistical Software*, *27*(8), 1–25. doi: 10.18637/jss.v027.i08
